# Impact of Geography and Rurality on Preconception Health Status in the United States

**DOI:** 10.5888/pcd20.230104

**Published:** 2023-11-09

**Authors:** Madison D. Haiman, Catherine Cubbin

**Affiliations:** 1 Steve Hicks School of Social Work, The University of Texas at Austin

## Abstract

**Introduction:**

Maternal illness and death are largely preventable; however, the field of preconception health needs further study. Geographic region and rurality play a large role in maternal health, and an understanding of the effect of these 2 factors at the individual level could prevent future adverse maternal health outcomes.

**Methods:**

We developed an abbreviated index of preconception health risk (diabetes, hypertension, body weight, mental health, unintended pregnancy, HIV, alcohol and nicotine use, nutrition, physical activity, receipt of the influenza vaccine) by using data from the 2019 Behavioral Risk Factor Surveillance System (BRFSS). A score of 1 was assigned for each behavior factor classified as unhealthy and a score of 0 for each factor classified as healthy, for a total potential score of 11. Respondent women from the 37 states that included the BRFSS family planning supplemental module who were aged 18 to 44 years who could become pregnant (N = 25,999) were included. We used univariate and multivariate regression models to assess the relationship between sociodemographic factors (age, race or ethnicity, relationship status, insurance status, education, income, and rurality and region) and preconception health, with a primary focus on rurality and region.

**Results:**

The average preconception health risk index score among participants was 3.5, with higher average scores in rural areas than in urban areas. All factors were independently associated with preconception health. Compared with women living in the urban Northeast, women living in all rural and region groups, except the rural West, had increased preconception health risk.

**Conclusion:**

Preconception health scores from our study showed that, on average, a person had more than 3 risk factors or behaviors. Given the current state of reproductive health policy in the United States, increased efforts are needed to address preconception health.

**Summary**

**What is already known on this topic?**

Maternal health is in crisis in the United States with many preventable maternal deaths. Preconception health influences maternal health.

**What is added by this report?**

Sociodemographic factors, particularly rurality and region, are related to preconception health. Our study was the first individual-level analysis of a preconception health-risk index for the United States.

**What are the implications for public health practice?**

The impact of social determinants of health on preconception health needs should be recognized and addressed along with preventive care programming in this understudied area.

## Introduction

Maternal health remains a significant problem in the United States. Awareness is increasing of the need for preconception health care — preventive health care before a person becomes pregnant — as a means of reducing the risk of maternal illness and death. The leading cause of pregnancy-related death from 2017 through 2019 was mental health conditions, followed by hemorrhage and pre-existing cardiac and coronary conditions (1). Both the first and third leading causes of death are linked to preconception health and are conditions that can be managed before pregnancy (1–3). Although prior studies in Canada (4) and Singapore (5) have used a composite index to examine preconception health risk among either their general populations (4) or those currently trying to conceive (5), studies have not examined this risk in the United States.

Prior research has pointed to geographic location, particularly rurality, as a social determinant of health that affects maternal illness and death, an area that needs further research (6). Geographic location determines place-based factors, such as access to transportation, housing, food, and health care and exposure to environmental conditions, such as violence and air and water quality, all of which can affect maternal health. Geographic location is important to examine in light of the disparities in maternal illness and death based on geographic location (6). Compared with pregnant urban dwellers with similar sociodemographic and medical backgrounds, pregnant rural dwellers have an increased risk of illness and death (7). Pregnant people who live in rural areas are 5 times more likely to live in a county that has a very high maternal vulnerability score compared with those residing in urban areas (8). People with preconception chronic conditions are more likely to reside in low-income rural areas than their urban counterparts (9). The South and Midwest have the highest levels of maternal vulnerability, indicating an increased risk for adverse birth outcomes in these regions (8). Measures, such as the maternal vulnerability index (8) that analyze systems-level preconception health indicators and studies that examine preconception health risk factors independently of one another (10,11) are both needed. However, an analysis is also needed at the individual level of composite preconception health status to better understand the health of women who make up these US counties, states, and regions. Our objective was to assess preconception health status in the United States and how rurality and regional residence affect it.

## Methods

### Data source

We analyzed cross-sectional data from the 2019 Behavioral Risk Factor Surveillance System (BRFSS) (12) to create a composite index to assess preconception health in rural areas. BRFSS is a national ongoing survey of noninstitutionalized adults conducted by state health departments in collaboration with the Centers for Disease Control and Prevention (12). Along with the general survey, which is distributed in all 50 states and participating territories, states and territories can elect to include various supplemental modules (12). Our analysis was limited to the 37 states that administered the family planning supplemental module to eligible respondents who were female, aged 18 to 49 years, not pregnant, and had not had a hysterectomy (N = 39,047). We excluded women aged 45 to 49 years (n = 8,397) and those who responded to the family planning module but indicated they were pregnant (n = 93) or used a permanent form of birth control (n = 3,953). Respondents with unknown or missing values for any of the covariates, except for income, were also excluded (n = 605). Respondents missing values for income (n = 3,560) were retained. Our final analytic sample totaled 25,999.

### Measures

We developed an abbreviated preconception health risk index (aPHRI) following recommendations from the American Academy of Family Physicians (AAFP) (2) and the American College of Obstetrics and Gynecology (ACOG) (3) for preconception health. The index included all indicators measurable through BRFSS data (13) (Table 1). For each measure of risk reported (either healthy [0] or unhealthy [1]), a score of 1 was added to the total score, for a potential total aPHRI score of 11. Any of these independent risks can harm a person’s reproductive health or potential future pregnancy; therefore, any respondent with a score above zero was considered to be at preconception health risk. A higher score indicated a higher preconception health risk. Spearman correlations between risk measures were all below 0.2.

**Table 1:** Components of the Abbreviated Preconception Health Risk Index, 2019 Behavioral Risk Factor Surveillance System (BRFSS), 37 states

Index component	BRFSS measure of risk
**Health factors**	
Pre-gestational diabetes	Told by a doctor, nurse, or other health professional they have diabetes, not including during pregnancy (3).
Pre-gestational hypertension	Told by a doctor, nurse, or other health professional they have high blood pressure, not including during pregnancy (3).
Weight	Body mass index (weight in kg divided by height in m^2^) score was other than normal (18.5–24.9), based on self-reported height and weight (2,3).
Mental health	Mental health was self reported to be “not good” 14 or more days in the last month (2,3).
**Health behaviors**	
Risk of unintended pregnancy	Currently sexually active, not using contraception, and not reporting any of the following: “don’t care if you get pregnant,” “you want a pregnancy,” or “same sex partner” (People who were currently pregnant or had a hysterectomy or tubal ligation were excluded from the study.) (2,3).
Risk of HIV and HIV screening	Answering yes when asked if any of the following situations apply to you: You have injected any drug other than those prescribed for you in the past year. You have been treated for a sexually transmitted disease in the past year. You have given or received money or drugs in exchange for sex in the past year. You had anal sex without a condom in the past year. You had 4 or more sex partners in the past year. And yes to the question, “Including fluid testing from your mouth, but not including tests you may have had for blood donation, have you ever been tested for HIV?” which indicates engaging in behaviors that put them at risk for contracting HIV in the past year and never tested for HIV (2,3).
Heavy alcohol use	Drinking more than 7 drinks in a week or 4 drinks in a single time period in the past 30 days (2,3).
Nicotine	Currently smoking cigarettes (2,3).
Nutrition	Eating fewer than 1.5 servings of fruit, or fewer than 2.5 servings of vegetables (if aged 31 or younger), or fewer than 2 servings of vegetables (if older than 31) daily (3,14).
Physical activity	Doing less than 150 min of physical activity in a week on average in the past month (3).
Influenza vaccine	Had not received an influenza vaccine in the past year (3).

We assigned states to regions based on the most recent census region classifications (15). The regional representation in this subsample was 14 states in the Southern region (Alabama, Arkansas, Delaware, Florida, Georgia, Louisiana, Maryland, Mississippi, North Carolina, Oklahoma, South Carolina, Tennessee, Virginia, and West Virginia), 5 in the Northeast region (Connecticut, Massachusetts, New York, Pennsylvania, and Rhode Island), 10 in the Midwest region (Illinois, Indiana, Iowa, Kansas, Minnesota, Missouri, Nebraska, Ohio, South Dakota, and Wisconsin), and 8 in the Western region (Arizona, Idaho, Hawaii, Montana, New Mexico, Oregon, Utah, and Wyoming). The regions were then further divided into rural and urban categories based on the BRFSS classification for a total of 8 rural regions (urban South, rural South, urban Northeast, rural Northeast, urban Midwest, rural Midwest, urban West, and rural West) (12).

We included sociodemographic and socioeconomic covariates in our analysis. The sociodemographic covariates were race or ethnicity (non-Hispanic American Indian or Alaska Native, non-Hispanic Asian, non-Hispanic Black, Hispanic, non-Hispanic White, non-Hispanic Native Hawaiian or Pacific Islander, non-Hispanic multiracial, non-Hispanic other race), annual household income from all sources (<$15,000; $15,000–$24,999; $25,000–$34,999; $35,000–$49,999; ≥$50,000, missing). Socioeconomic covariates were insurance status (insured or uninsured), age (18–24 y, 25–34 y, 35–44 y), education status (did not graduate from high school, graduated from high school, attended college or technical school, graduated from college or technical school), and relationship status (in a married or unmarried relationship or not in a relationship).

### Analysis

We followed univariate and bivariate analyses with linear regression. Models were built sequentially. First, each covariate was included, one at a time (unadjusted models); then, all covariates except for socioeconomic factors (insurance status, education, and income) were included (sociodemographic model). Last, a final model was run that included all covariates. Significance was determined by a value of *P* < .05. We used SAS software version 9.4 (SAS Institute Inc) for all data analyses. All analyses were weighted and adjusted for the complex sample design.

## Results

Only 6.5% of respondents lived in rural areas (Table 2), and the highest proportion of those rural women lived in the South (45.3%) versus the Northeast (6.4%). Rural areas in all geographic regions showed higher aPHRI scores than their urban counterparts, and geographic regions differed, with the highest means in the South and Midwest and the lowest in the Northeast.

**Table 2:** Characteristics of People Who May be Able to Become Pregnant and Adjusted Mean Abbreviated Preconception Health Risk Index (aPHRI) Scores, Behavioral Risk Factor Surveillance System, 2019, N = 25,999

Characteristic	N (%)^a^	Mean aPHRI Score (SE)
**Total population**		
Urban	22,881 (93.5)	3.53 (0.01)
Rural	3,118 (6.5)	3.80 (0.03)
**Rurality and region**		
South	8,752 (39.1)	3.67 (0.02)
Urban	7,728 (92.5)	3.65 (0.02)
Rural	1,024 (7.5)	3.93 (0.05)
Northeast	3,038 (22.2)	3.34 (0.03)
Urban	2,992 (98.1)	3.33 (0.03)
Rural	46 (1.9)	3.89 (0.22)
Midwest	8,403 (28.5)	3.58 (0.02)
Urban	7,027 (91.0)	3.57 (0.02)
Rural	1,376 (9.0)	3.69 (0.04)
West	5,806 (10.3)	3.42 (0.02)
Urban	5,134 (93.5)	3.41 (0.02)
Rural	672 (6.5)	3.61 (0.06)
**Age, y**		
18–24	5,358 (29.1)	3.40 (0.02)
25–34	9,547 (36.9)	3.61 (0.02)
35–44	11,094 (33.9)	3.61 (0.01)
**Race or ethnicity**		
Non-Hispanic American Indian or Alaska Native	545 (1.0)	4.01 (0.07)
Non-Hispanic Asian	830 (4.7)	2.96 (0.05)
Non-Hispanic Black	3,002 (17.0)	3.88 (0.03)
Hispanic	3,383 (15.8)	3.55 (0.02)
Non-Hispanic White	17,053 (58.7)	3.48 (0.01)
Non-Hispanic multiracial	851 (2.1)	3.77 (0.05)
Non-Hispanic Native Hawaiian or Pacific Islander	198 (0.5)	3.98 (0.11)
Non-Hispanic other race	137 (0.3)	3.81 (0.15)
**Relationship status**		
In a married or unmarried relationship	12,402 (47.4)	3.41 (0.01)
Not in a relationship	13,597 (52.6)	3.67 (0.01)
**Insurance status**		
Insured	22,358 (84.8)	3.48 (0.01)
Uninsured	3,641 (15.2)	3.91 (0.02)
**Education**		
Did not graduate high school	1,527 (9.8)	3.91 (0.04)
Graduated high school	5,805 (25.7)	3.80 (0.02)
Attended college/technical school	7,961 (34.0)	3.66 (0.02)
Graduated college/technical school	10,706 (30.6)	3.10 (0.01)
**Annual income from all sources, $**		
<15,000	2,122 (8.6)	4.04 (0.03)
15,000–24,999	3,606 (14.7)	4.01 (0.02)
25,000–34,999	2,158 (8.8)	3.79 (0.03)
35,000–49,999	3,126 (11.8)	3.77 (0.03)
≥50,000	11,427 (40.0)	3.23 (0.01)
Missing	3,560 (16.2)	3.38 (0.02)

a Percentages are weighted.

The largest age group was women aged 25 to 34 years (36.9%) and the largest racial or ethnic group was non-Hispanic White (58.7%). Of all other racial and ethnic groups, 17.0% of participants identified as non-Hispanic Black, 15.8% identified as Hispanic, 4.7% identified as non-Hispanic Asian, and 6.6% or less were from the remaining groups (non-Hispanic American Indian or Alaska Native, non-Hispanic multiracial, non-Hispanic Native Hawaiian or Pacific Islander, and non-Hispanic other race). Relationship status was nearly evenly distributed, and most respondents were insured (84.8%). Of the respondents, 34.0% attended some college or technical school, and 30.6% reported graduating from college or technical school; only 9.8% had not graduated from high school. Forty percent reported an annual household income of $50,000 or more, and 22.3% had an annual income less than $25,000. Income was missing for 16.2%.

Women aged 25–44 years had higher aPHRI scores than women aged 18–24. By race or ethnicity, respondent aPHRI scores were as follows: non-Hispanic American Indian, 4.01; non-Hispanic Black, 3.88; non-Hispanic multiracial, 3.77; non-Hispanic Native Hawaiian or Pacific Islander, 3.98; and non-Hispanic other race, 3.81. All the foregoing had higher mean aPHRI scores than participants who identified as Hispanic, 3.55; non-Hispanic White, 3.48, or non-Hispanic Asian, 2.96. Respondents who were not in a relationship (3.67) or were uninsured (3.91) had a higher mean aPHRI score than those in a relationship or insured. Respondents with lower incomes or education had higher aPHRI scores than those with higher incomes or education.

The most prevalent preconception health risk factors and behaviors in the total sample by aPHRI score were poor nutrition (87.0%), not having had an influenza vaccination (60.5%), and unhealthy weight (55.9%) (Table 3). This was consistent across all rurality and region groups except for the rural Northeast, where the third most prevalent component was risk of unintended pregnancy (53.2%) compared with 28.6% overall. In most cases, the prevalence of each component was higher among rural women than urban women in each region. The greatest disparities in prevalence by rurality and region were related to risk of unintended pregnancy between the urban West and rural Northeast (25.4% vs 53.2%), nicotine use between the urban West and rural Northeast (11.6% vs 37.1%), and heavy alcohol use (drinking more than 7 drinks in a week or 4 drinks on a single occasion in the past 30 days) between the rural South and the urban Midwest (12.2% vs 24.5%).

**Table 3:** Prevalence of Abbreviated Preconception Health Risk Index Factors and Behaviors, by Rurality and Region Group, Among Participants (N = 25,999), Behavioral Risk Factor Surveillance System, 2019^a^

Risk factor	Total population prevalence, %	South		Northeast		Midwest		West	
		Urban	Rural	Urban	Rural	Urban	Rural	Urban	Rural
Pregestational diabetes	3.0	3.5	4.1	2.4	0.0	2.6	2.5	3.5	2.8
Pregestational hypertension	11.0	13.5	13.4	8.8	9.6	9.7	7.7	9.5	10.0
Unhealthy weight^b^	55.9	57.5	66.8	52.4	49.6	56.1	58.4	54.0	56.2
Poor mental health	19.6	20.1	19.9	17.8	26.6	20.2	20.1	20.2	19.9
Risk of unintended pregnancy	28.6	29.8	36.6	27.0	53.2	27.9	32.6	25.4	30.9
Risk of HIV, HIV screening^c^	2.8	2.9	2.7	2.6	1.9	3.0	1.5	3.4	2.9
Heavy alcohol use^d^	20.6	19.3	12.2	21.0	20.1	24.5	18.3	17.5	19.0
Nicotine use^e^	14.8	15.1	23.8	12.7	37.1	15.2	22.3	11.6	17.2
Poor nutrition^f^	87.0	87.4	89.5	84.7	86.4	88.1	86.2	86.8	89.2
Poor physical activity^g^	51.0	51.1	54.5	50.2	49.5	52.8	53.3	46.3	43.6
No influenza vaccine	60.5	65.2	66.7	53.6	55.3	57.5	66.1	63.3	68.8

a Values are percentages.

b Body mass index (weight in kg divided by height in m^2^) score other than normal (18.5–24.9) based on self-reported height and weight.

c Answering yes when asked if any of the following situations apply: have injected any drug other than those prescribed in the past year, have been treated for a sexually transmitted disease in the past year, have given or received money or drugs in exchange for sex in the past year, had anal sex without a condom in the past year, had 4 or more sex partners in the past year, and answering yes to the question, “Including fluid testing from your mouth, but not including tests you may have had for blood donation, have you ever been tested for HIV?” which indicates engaging in behaviors that put a person at risk for contracting HIV in the past year but never tested for HIV (2,3).

d Drinking more than 7 alcoholic drinks in a week or 4 drinks on a single occasion in the past 30 days (2,3).

e Currently smoking cigarettes (2,3).

f Eating fewer than 1.5 servings of fruit or 2.5 servings of vegetables daily (if aged 31 years or younger), or fewer than 2 servings of vegetables daily if older than 31 years (3,14).

g Doing less than 150 min of physical activity on average in a week in the past month (3).

All variables were significant when run in separate regression models examining composite aPHRI scores (Table 4). By rurality and region, when compared with residing in the urban Northeast, women residing in all other areas except for the urban West or rural Northeast had significantly higher aPHRI scores. By race and ethnicity, when compared with non-Hispanic White, American Indian or Alaska Native, Black, multiracial, and non-Hispanic Native Hawaiian or Pacific Islander women had significantly higher aPHRI scores. Non-Hispanic Asian women had significantly lower scores. Women aged 18 to 24 years had significantly lower aPHRI scores than women aged 35 to 44 years, and women who were not in a relationship had higher aPHRI scores than women in a relationship. Women with lower incomes or education or who were not insured also had significantly higher aPHRI scores than their respective reference groups. Results were generally similar in the sociodemographic model. In the final model, including socioeconomic variables, being non-Hispanic and residing in the rural West or self-identified as multiracial were no longer significant. Hispanic women had significantly lower aPHRI scores compared with non-Hispanic White women. All socioeconomic variables, except for missing income data, remained significantly associated with aPHRI score.

**Table 4:** Associations Between Demographic and Socioeconomic Factors and Abbreviated Preconception Health Risk (aPHRI) Index, Behavioral Risk Factor Surveillance System, 2019, N = 25,999

Demographic	Unadjusted models		Sociodemograhic model		Final model	
	Beta (SE)	*P* value^a^	Beta (SE)	*P* value^b^	Beta (SE)	*P* value^c^
Rurality and region						
Urban Southeast	.104 (.045)	<.001	.082 (.052)	<.001	.060 (.050)	<.001
Rural Southeast	.068 (.078)	<.001	.062 (.077)	<.001	.039 (.076)	<.001
Urban Northeast	Reference					
Rural Northeast	.024 (.291)	.05	.024 (.271)	.04	.018 (.248)	.09
Urban Midwest	.072 (.053)	<.001	.072 (.052)	<.001	.053 (.050)	<.001
Rural Midwest	.038 (.083)	<.001	.043 (.082)	<.001	.027 (.078)	.001
Urban West	.016 (.057)	.15	.017 (.057)	.12	.006 (.055)	.60
Rural West	.014 (.102)	.007	.012 (.102)	.02	.005 (.101)	.36
Age, y						
18–24	–.065 (.042)	<.001	–.113 (.044)	<.001	–.154 (.045)	<.001
25–34	–.000 (.037)	.98	–.018 (.036)	.13	–.040 (.035)	<.001
35–44	Reference					
Race or ethnicity						
Non-Hispanic American Indian	.036 (.124)	<.001	.032 (.129)	<.001	.016 (.120)	.05
Non-Hispanic Asian	.075 (.102)	<.001	.069 (.099)	<.001	.055 (.094)	<.001
Non-Hispanic Black	.101 (.047)	<.001	.076 (.049)	<.001	.046 (.047)	<.001
Hispanic	.017 (.049)	.15	.022 (.049)	.07	.047 (.052)	<.001
Non-Hispanic multiracial	.027 (.126)	.02	.027 (.120)	.02	.021 (.117)	.06
Non-Hispanic Native Hawaiian or Pacific Islander	.017 (.214)	.02	.019 (.209)	<.001	.014 (.194)	.04
Non-Hispanic White	Reference					
Non-Hispanic other race only	.015 (.227)	.14	.011 (.230)	.28	.009 (.221)	.35
Relationship status						
In a relationship	Reference					
Not in a relationship	.086 (.033)	<.001	.113 (.035)	<.001	.069 (.036)	<.001
Insurance status						
Insured	Reference		Not included in model		Reference	
Not Insured	.103 (.046)	<.001			.049 (.047)	<.001
Education status						
Did not graduate high school	.163 (.068)	<.001	Not included in model		.123 (.072)	<.001
Graduated high school	.205 (.042)	<.001			.166 (.045)	<.001
Attended college or technical school	.179 (.037)	<.001			.148 (.039)	<.001
Graduated college or technical school	Reference				Reference	
Annual income, $						
<15,000	.153 (.059)	<.001	Not included in model		.092 (.066)	<.001
15,000 to 24,999	.185 (.048)	<.001			.118 (.054)	<.001
25,000 to 34,999	.107 (.058)	<.001			.064 (.062)	<.001
35,000 to 49,999	.117 (.054)	<.001			.085 (.054)	<.001
≥50,000	Reference				Reference	
Income missing	.038 (.048)	.002			.002 (.054)	.87

a *P* values were determined from univariate linear regression analyses of composite aPHRI scores.

b *P* values were determined from the sociodemographic multivariate regression analysis of composite aPHRI scores, excluding socioeconomic factors.

c *P* values were determined from the final multivariate regression analysis or composite aPHRI scores including socioeconomic factors.

## Discussion

As the first composite, individual-level measurement of preconception health in the United States, our study assesses its status. Although prior studies have analyzed preconception health factors independently (10,11) or at a community level (5), no study has examined cumulative, individual-level risk factors. The aPHRI scores in our overall study sample, and particularly the scores found in rural areas, show the need to address preconception health issues to prevent or reduce adverse maternal health outcomes. The aPHRI score reflects the number of risk factors and behaviors an individual has, meaning that on average a woman in the United States has 3.6 risk factors or behaviors that could put her at increased risk for adverse maternal health outcomes, particularly residents of rural areas. It should be noted that only 6.5% of our sample resided in a rural area.

In addition to the already high mean aPHRI score, especially in a relatively young population-based sample, large disparities occurred in mean aPHRI scores across all sociodemographic and socioeconomic factors analyzed. Disparities existed by rurality and region and by age, race or ethnicity, relationship status, insurance status, education, and income, with the lowest mean scores among non-Hispanic Asian women (2.96) and the highest among women with an annual household income lower than $15,000 (4.06). Furthermore, all variables were found to be independently associated with the aPHRI score, building on existing literature that has shown that rurality, region, race and ethnicity, age, and insurance status all affect preconception (8,16) and maternal health (6–8). Historically, social determinants of health have been linked to access to and receipt of adequate health care (17), and this extends to preconception health. Prevention efforts should recognize and address the impact of social determinants of health on preconception health and, ultimately, maternal health.

The South and Midwest had the highest mean aPHRI scores, and both the South (8,16) and Midwest (8) have previously been identified as having worse preconception health than other regions. This comes as no surprise, because all but 2 of the states we studied in these regions had expanded Medicaid at the time the data used in this analysis were collected. Seven of the 12 Southern states and 7 of the 12 Midwestern states included in our analysis did not have expanded Medicaid and had the highest average aPHRI scores (18). We found a significant association between insurance status and aPHRI score. Private health care practices and clinics could include preconception health screenings and interventions during routine general and women’s health appointments (19), particularly preconception health interventions that are culturally appropriate and address geographic, cultural, and sociodemographic differences (20). Recognizing that the groups at highest preconception health risk are often those without access to consistent health care, large systems-level changes need to be made.

More than three-quarters of our sample population had poor nutrition, and more than half had an unhealthy body weight or had not received an influenza vaccination. This was consistent in all rural and urban areas except the rural Northeast where the risk of unintended pregnancy was the third most prevalent aPHRI factor. An estimated 69% of US women do not fall within a healthy range according to the body mass index (weight in kg divided by height in m^2^) (21), and an increase in the prevalence of pre-pregnancy obesity has been reported (22). Only 36% of adults aged 18 to 49 years received an influenza vaccination in the 2022–2023 season (23). Overall, 14.5% of women met the daily fruit consumption recommendations in 2019, and 12.4% met the daily recommendations for vegetables (24). These numbers are all within a range similar to what we found in our study. All the factors and behaviors our study analyzed were based on ACOG and AAFP assessments, which indicates that unhealthy preconception status leads to increased risk for adverse maternal health outcomes (7,8). 

Across all groups, the lowest prevalence of risk, 25.4% for unintended pregnancy, was in the urban West, indicating that across all rurality and region groups, at least 1 in 4 women were at risk for an unintended pregnancy. However, all rural groups had a higher prevalence of unintended pregnancy compared with urban groups where nearly 1 in 3 women were at risk for an unintended pregnancy. The prevalence of risk for unintended pregnancy in the overall sample population was 28.6%. These high rates further emphasize the critical need to address preconception health risk factors and behaviors before a person becomes pregnant to prevent adverse maternal health outcomes, particularly in rural areas. In 2017, when there was still a federal legal right to abortion in the United States, state-level data indicated that 16% to 59% of unwanted pregnancies were terminated (25). With federal protection no longer in place and abortion illegal in nearly half of all states, more unwanted pregnancies are being carried to term (26). The results of this study add to the existing literature to emphasize the need to address the concerning rates of preconception health in the United States as a whole (7,8), and specifically in rural areas (5). Consistent with prior literature, rural residents in our study had high rates of preconception health risks (27). Given the current status of reproductive health policy in the United States, the high number of our study participants at risk for an unintended pregnancy, the high aPHRI scores overall found in this study, and the impact of sociodemographic and socioeconomic factors on aPHRI scores, increased preventive preconception health care is needed now (28).

### Limitations

Our study had several limitations. First, the data used were self-reported. Second, the stigma surrounding mental health conditions and substance use likely caused participants to underreport their mental health status and nicotine and alcohol use. However, despite assumed underreporting, we found a high prevalence of these 3 factors. Third, the awareness of conditions such as diabetes and hypertension rely on a medical diagnosis and are contingent on a person having access to appropriate medical care. This likely contributed to underreporting of pregestational diabetes and hypertension seen in our results, particularly in rural areas. Prior studies have estimated the prevalence of pregestational hypertension and diabetes among women of reproductive age to be 9.3% and 4.5%, respectively, with nearly 17% of those with hypertension and 30% of those with diabetes unaware of their condition (29). Fourth, our results are not generalizable to all women. Our study was limited to 37 states that completed the BRFSS family planning module. BRFSS also had varying response rates by state. Complex survey weights were employed to increase generalizability. Most of the youngest women in our sample would not have had the opportunity to complete the highest level of education we measured. Lastly, although the measures included in aPHRI have been linked to maternal health outcomes, the index as a whole has not been validated. A validated composite measure of preconception health is needed to examine preconception health status in the United States over time. Despite these limitations, our study illustrates the relationship between sociodemographic and socioeconomic factors and preconception health.

### Conclusion

Our aPHRI analysis provides an overview of the current state of preconception health in the United States and the differences that exist by rurality and region. This overview is especially timely given the recent restriction to reproductive health care resulting from the Dobbs vs Jackson Women’s Health Organization decision, removing the constitutional right to abortion (30). It is more critical now than ever to address preconception health in an effort to prevent adverse maternal health outcomes. To address the preconception health needs of people able to become pregnant continued examination of best practices is needed to consider rural and geographic group status, along with other social determinants of health, specifically increasing access to health care. Although our study examines preconception health, which is focused on the health of people who may be able to become pregnant, preconception health is only one component of reproductive health. Reproductive health as a whole should be available to all people, regardless of the desire to become pregnant or fertility status.
